# Association between Proton Pump Inhibitors and Hearing Impairment: A Nested Case-Control Study

**DOI:** 10.3390/cimb43010012

**Published:** 2021-05-18

**Authors:** So Young Kim, Chang Ho Lee, Chanyang Min, Dae Myoung Yoo, Hyo Geun Choi

**Affiliations:** 1 Department of Otorhinolaryngology-Head & Neck Surgery, CHA Bundang Medical Center, CHA University, Seongnam 13496, Korea; sossi81@hanmail.net (S.Y.K.); hearwell@gmail.com (C.H.L.); 2 Hallym Data Science Laboratory, Hallym University College of Medicine, Anyang 14068, Korea; joicemin@naver.com (C.M.); ydm1285@naver.com (D.M.Y.); 3 Graduate School of Public Health, Seoul National University, Seoul 08826, Korea; 4 Department of Otorhinolaryngology-Head & Neck Surgery, Hallym University College of Medicine, Anyang 14068, Korea

**Keywords:** hearing loss, proton pump inhibitors, case-control studies, cohort studies, epidemiology

## Abstract

This study investigated the association of previous use of proton pump inhibitors (PPIs) with the rate of hearing impairment. The ≥40-year-old population in the Korean National Health Insurance Service-Health Screening Cohort was enrolled. The 6626 registered hearing-impaired patients were matched with 508,240 control participants for age, sex, income, region of residence, and index date (date of hearing impairment diagnosis). The prescription histories of PPIs were collected for 2 years before the index date. The odds ratios of the duration of PPI use for hearing impairment were analyzed using conditional logistic regression. Subgroups of age/sex and severity of hearing impairments were additionally analyzed for the relation of PPI use with hearing impairment. PPI use for 30–365 days was associated with a 1.65-times higher odds of hearing impairment (95% confidence interval (CI) = 1.47–1.86 for 30–365 days of PPI medication). PPI use for ≥365 days was also related to 1.52-times higher odds of hearing impairment (95% CI = 1.35–1.72, *p* < 0.001). All age and sex subgroups demonstrated a positive association between PPI use and hearing impairment. Severe hearing impairment showed consistently higher odds of a relation with PPI use. PPI use was associated with an increased rate of hearing impairment.

## 1. Introduction

Proton pump inhibitors (PPIs) have been widely used for the treatment of several acid-related disorders, such as gastroesophageal reflux disease (GERD), gastric ulcer, duodenal ulcer, erosive esophagitis, and laryngeal reflux diseases [1,2]. PPIs act as antacids by irreversibly inhibiting H+/K+ ATPase [3]. Because PPIs have been shown to have superior remedial effects than other antacids, such as histamine-2 receptor antagonists (H2RAs) [4], the prevalence of PPI prescriptions has reached approximately 2.9–7.8% in the middle-aged adult population in the US [5]. However, an increasing number of researchers have suggested the possibility of adverse effects of PPIs [6,7]. In addition to mild side effects, such as diarrhea, nausea, vomiting, and headache, several recent studies have reported electrolyte imbalance [6], kidney injury [7], and dementia [8] as possible side effects. PPIs modify pH, which may impede protease and lysosomal activities. These protease and lysosomal dysfunctions could result in beta-amyloid accumulation and neural degeneration [8]. Additionally, a retrospective study on adverse event reports demonstrated a higher rate of neurologic side effects, including cognitive dysfunction, vision loss, and hearing impairment [5].

Hearing impairment is one of the most common sensorineural disorders worldwide, affecting approximately 6.1% of the world’s population [9]. The etiologies of hearing impairment are complex, but more than 70% of sensorineural hearing impairment has been known to be associated with cochlear dysfunction. Because the cochlea is supplied by the labyrinthine artery without collateral blood supply and is an oxygen-demanding organ, it is vulnerable to ischemic insults, such as those caused by ototoxic drugs, noise exposure, and the aging processes [10,11,12]. In addition, the cochlear endolymphatic potential generates and regulates mechanoelectrical signal transduction from outer hair cells to spiral ganglion cells, and electrical imbalance and perturbation of endolymph vs. perilymph homeostasis could result in hearing impairment [13].

Because PPIs have adverse impacts on neurologic disorders via ischemia and electric imbalance, adverse effects of PPIs on cochlear function could be predicted [6,14]. Therefore, we hypothesized that the prolonged use of PPIs could increase the occurrence of hearing impairment. To evaluate this hypothesis, hearing impairment patients were evaluated for the previous use of PPIs and were compared to the matched control group. In addition, confounders for hearing impairment, such as smoking and comorbidities of cardiovascular and neurologic diseases, were considered.

## 2. Materials and Methods

### 2.1. Ethics

The present study was approved by the ethics committee of Hallym University (2019-10-023: approval date: 5 November 2019). The requirement for written informed consent was exempted by the ethics committee of Hallym University. All studies were conducted according to the guidelines and regulations of the ethics committee of Hallym University.

### 2.2. Study Population and Participant Selection

**Figure 1:**
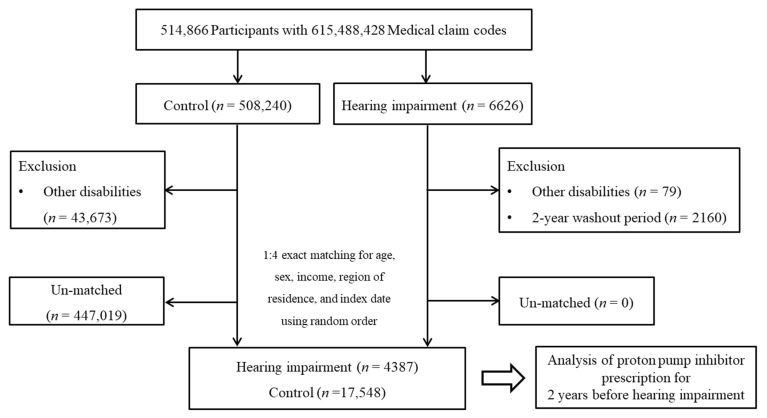
A schematic illustration of the participant selection process that was used in the present study.

This study used Korean National Health Insurance Service-Health Screening Cohort data [15]. Participants with hearing impairment were selected from 514,866 participants with 615,488,428 medical claim codes from 2002 through 2015 (*n* = 6626). Participants were included in the control group if they were not defined as having hearing impairment from 2002 through 2015 (*n* = 508,240). Participants who were diagnosed with other disabilities were excluded (*n* = 79 for hearing impairment participants, *n* = 43,673 for control participants). To measure PPI history in the previous 2 years, we excluded participants with hearing impairment who were diagnosed with hearing impairment before 2003 (*n* = 2160). Hearing impairment participants were 1:4 matched with control participants for age, sex, income, and region of residence. The control participants were randomly selected. The date of hearing impairment diagnosis was defined as the index date, and the same index date was used for the matched control participants. The 447,019 control participants whose index date did not match that of the hearing impairment participants were excluded. A total of 4387 participants with hearing impairment and 17,548 control participants were enrolled (Figure 1).

### 2.3. Exposure (Days of Proton Pump Inhibitor Prescription)

The days of PPI prescription were defined as the total prescription days during the 2 years before the index date. Prescription days of PPIs were categorized as <30 days, ≥30 to <365 days, and ≤365 days. To prevent duplicate prescriptions of PPIs, of the prescription days that started on the same day, only the longest prescription duration was included.

### 2.4. Outcome (Hearing Impairment)

Participants with hearing impairment who were registered as having hearing impairment by the Ministry of Health and Welfare were selected. Participants who had other disabilities were excluded. According to the degree of hearing impairment, severe hearing impairment was classified as hearing thresholds of ≥60 dB in both ears or hearing thresholds of ≥80 dB in one ear and ≥40 dB in one ear. Profound hearing impairment was classified as a hearing threshold of ≥90 dB in both ears [16]. All hearing impairment participants underwent three pure-tone audiometry tests (PTAs) and auditory brainstem responses [16].

### 2.5. Covariates

Age groups were classified into 5-year intervals. Income groups were divided into 5 classes (class 1 (lowest income) to 5 (highest income)). The region of residence was classified as urban or rural [17]. Tobacco smoking, alcohol consumption, and obesity according to body mass index (BMI, kg/m^2^) were categorized as previously described [18]. The records of total cholesterol (mg/dL), systolic blood pressure (SBP, mmHg), diastolic blood pressure (DBP, mmHg), and fasting blood glucose (mg/dL) were used. Missing fasting blood glucose (*n* = 2 (0.009%)) and total cholesterol (*n* = 3 (0.013%)) values were substituted by the average values of the study participants.

The Charlson Comorbidity Index (CCI) was calculated for 17 comorbidities as a continuous variable (0 (no comorbidities) through 29 (multiple comorbidities)) [19]. Dementia was not included in the CCI score.

Regarding PPIs, the number of patients diagnosed with GERD (ICD-10 code: K21, treated ≥2 times and prescribed a PPI for ≥2 weeks) and the dates of H2 blocker prescription were additionally assessed. The number of patients diagnosed with GERD and the prescription dates of H2 blockers were assessed for the 2 years prior to the index date.

### 2.6. Statistical Analyses

The hearing impairment and control groups were compared using the chi-square test for categorical variables and the independent *t* test for continuous variables.

Conditional logistic regression analysis was conducted, and the odds ratios (ORs) and 95% confidence intervals (Cis) of the prescription days of PPIs for hearing impairment were calculated. A crude model (simple), model 1 (SBP, DBP, fasting blood glucose, and total cholesterol), model 2 (model 1 plus obesity, smoking, alcohol consumption, and CCI scores), and model 3 (model 2 plus gastroesophageal reflux disease and H2 blocker) were used. The matched variables were stratified.

Age and sex (<70 years old and ≥70 years old, men and women) subgroups were analyzed. We further analyzed the ORs of proton pump inhibitor prescription days for hearing impairment according to severity of hearing impairment. Two-tailed analyses were performed. *p*-values less than 0.05 were defined as statistically significant. Statistical analyses were performed using SAS version 9.4 (SAS Institute Inc., Cary, NC, USA).

## 3. Results

The prescription days of PPI were different between the hearing impairment and control groups (10.3% vs. 15.3% of <30 days of PPI prescription, *p* < 0.001, Table 1). The hearing impairment group showed differences in the rate of alcohol consumption, SBP, DBP, fasting blood glucose, CCI score, gastroesophageal reflux disease, and H2 blocker use (all *p* < 0.05).

The participants with prior histories of PPI prescription demonstrated higher odds of hearing impairment (Table 2). The ≥30 to <365 days of PPI prescription was related with 1.65 higher odds for hearing impairment (95% CI = 1.47–1.86, *p* < 0.001). The ≥365 days of PPI prescription was associated with 1.52 higher odds for hearing impairment (95% CI = 1.35–1.72, *p* < 0.001).

The previous histories of PPI prescription were associated with higher odds for hearing impairment in all age and sex subgroups. The <70-year-old group who had a PPI prescription for ≥30 to <365 days showed 1.70 (95% CI = 1.49–1.95) higher odds of hearing impairment than those with a PPI prescription for ≥0 to <30 days. The men’s group who had a PPI prescription of ≥30 to <365 days showed 1.80 (95%CI = 1.57–2.07) higher odds of hearing impairment than those with a PPI prescription for ≥0 to <30 days.

According to the degree of hearing impairment, the severe hearing impairment group, but not the profound hearing impairment group, exhibited a relationship between a longer duration of PPI prescription and a higher rate of hearing impairment (Table 3).

## 4. Discussion

The long-term use of PPIs was linked with an increased rate of hearing impairment in the adult population. This relation of PPI with hearing impairment was maintained in all age and sex subgroups. This is a pioneering study on the potential impacts of PPI use on hearing impairment in a large population. We comprehensively considered possible confounders, such as lifestyle factors of smoking and alcohol consumption and comorbidities.

Two previous studies suggested the association of PPIs with hearing impairment. The National Health and Nutrition Examination Survey study on the adverse effects of PPI revealed significantly increased risks of hearing impairment, dementia, migraine, and other peripheral neuropathies associated with PPI use [5]. However, this study had limitations because it was based on the adverse effects of PPI use. Another prospective cohort study in middle-aged women investigated the association of PPI use with self-reported hearing impairment [20]. Although PPI use was not associated with self-reported hearing impairment in that study after adjusting for GERD symptoms, that study did not objectively measure either hearing impairment or GERD. All variables, including PPI use, were based on self-reported survey items, which limited the fidelity of their data. The present study objectively measured PTAs, and prescription data of PPIs were collected, thereby improving the fidelity of the data. In addition, prior studies have reported the association of long-term PPI use with dementia and sensory disorders, such as vision and smell losses [5,21]. Compared to histamine−2 receptor antagonist, PPI use was related with increased propensity for adverse neurological effects, including migraine, severe peripheral neuropathies, and visual abnormalities [5]. A few plausible pathophysiological mechanisms could link PPI use with hearing impairment.

Insufficient blood supply could mediate ischemic injury in the inner ear [22]. PPIs inhibit endothelial nitric oxide synthetase, which reduces nitric oxide in circulation [14]. Vascular endothelium-derived nitric oxide is essential for the regulation of vasodilation, platelet adhesion/aggregation, and antiatherosclerotic and anti-inflammatory effects [23]. Thus, the decreased level of endothelium-derived nitric oxide could increase ischemic injury and oxidative stress. In addition, PPI is known as a competitive inhibitor of cytochrome CYP2C19, which also interacts with clopidogrel [24]. The inhibition of the efficacy of clopidogrel could increase the risk of thromboembolism and coronary syndrome [25]. Because of impaired vasodilation and ischemic changes associated with PPIs, a number of previous studies have demonstrated an increased risk of cardiovascular diseases related to PPI use [26,27]. A review study described that PPI use was related to excess mortality from cardiovascular diseases in 15/1000 persons [27]. As the cochlea is susceptible to ischemic injury due to the high oxygen demands and blood supply from the end artery [10,11], ischemia following PPI use could impact cochlear dysfunction and hearing impairment.

Metabolic disturbances and the malabsorption of micronutrients may induce neuronal degeneration. A number of prior studies suggested that the metabolic disturbances associated with PPI use were linked with an increased risk of dementia [8,28]. The modulation of protease activities due to changes in pH resulting from PPI use could induce the accumulation of beta-amyloid [29]. In addition, inhibition of lysosomal activities due to the inhibition of vacuolar H+-ATPase was suggested to decrease the clearance of beta-amyloid peptides. Because dementia and neuronal degeneration have been linked with neural presbycusis-type hearing impairment, these metabolic changes could increase the risk of hearing impairment [30]. Moreover, PPIs interfere with the absorption of micronutrients, such as iron, vitamin B12, and nitric oxide [31]. The malabsorption of micronutrients has been reported to be related to cochlear dysfunction [32].

The dysfunction of H, K-ATPase in the cochlear lateral wall could induce electrical imbalance and disturb homeostasis of the endolymphatic fluid of the cochlea. It was reported that a proton pump identical to that in the stomach is expressed in the lateral wall of the cochlea [33]. The cochlear proton pump was suggested to play a crucial role in maintaining a high potassium ion concentration in the endolymph, which sustains the cochlear endolymphatic potential [34]. PPI use could inhibit the cochlear proton pump as well as the gastric proton pump, dysregulating the inner ear potential and mediating hearing impairment.

This study used data from a large nationwide representative cohort. Many control participants could be selected and matched for demographic and socioeconomic factors. The degree of hearing loss was based on three PTAs tests and auditory brainstem response test results. The use of multiple objective hearing measures prevented the misdiagnosis of hearing impairment in this study. Because registered hearing-impaired persons receive support for the cost of health care, including hearing aids, most hearing-impaired persons were included in our hearing-impaired group. However, some limitations should be considered when interpreting the current results. The degree and etiologies of hearing loss could not be detailed in this study. For histories of PPI prescriptions, the types and doses of PPIs could not be differentiated in the current data. Although past medical histories, including history of GERD, were adjusted, the potential confounding effects of reflux symptoms remained. Further studies on the associations of dose and types of PPI with the specific types of hearing loss will unravel the current questions. The analyses using machine learning approach could facilitate the manipulations of huge data.

## 5. Conclusions

Longer durations of PPI use were related to hearing impairment in adult Koreans. This relation of PPIs with hearing impairment was valid in all age and sex groups. Therefore, patients who need long-term PPI medication should be consulted for their hearing preservation. Future study with randomized controlled trial study design could delineate the causal relation between PPI use and hearing impairment.

## Figures and Tables

**Table 1:** General characteristics of participants.

Characteristics	Total Participants		
	Hearing Impairment (*n*, %)	Control (*n*, %)	*p*-Value
Age (years old)			1.000
40–44	42 (1.0)	168 (1.0)	
45–49	144 (3.3)	576 (3.3)	
50–54	302 (6.9)	1208 (6.9)	
55–59	485 (11.1)	1940 (11.1)	
60–64	621 (14.2)	2484 (14.2)	
65–69	788 (18.0)	3152 (18.0)	
70–74	851 (19.4)	3404 (19.4)	
75–79	715 (16.3)	2860 (16.3)	
80–84	356 (8.1)	1424 (8.1)	
85+	83 (1.9)	332 (1.9)	
Sex			1.000
Male	2651 (60.4)	10,604 (60.4)	
Female	1736 (39.6)	6944 (39.6)	
Income			1.000
1 (lowest)	829 (18.9)	3316 (18.9)	
2	612 (14.0)	2448 (14.0)	
3	693 (15.8)	2772 (15.8)	
4	809 (18.4)	3236 (18.4)	
5 (highest)	1444 (32.9)	5776 (32.9)	
Region of residence			1.000
Urban	1807 (41.2)	7228 (41.2)	
Rural	2580 (58.8)	10,320 (58.8)	
Obesity ^‡^			0.457
Underweight	141 (3.2)	623 (3.6)	
Normal	1611 (36.7)	6399 (36.5)	
Overweight	1145 (26.1)	4735 (27.0)	
Obese I	1382 (31.5)	5342 (30.4)	
Obese II	108 (2.5)	449 (2.6)	
Smoking status			0.097
Nonsmoker	3182 (72.5)	12,439 (70.9)	
Past smoker	477 (10.9)	2031 (11.6)	
Current smoker	728 (16.6)	3078 (17.5)	
Alcohol consumption			
<1 time a week	3234 (73.7)	12,640 (72.0)	0.025 *
≥1 time a week	1153 (26.3)	4908 (28.0)	
Systolic blood pressure			<0.001 *
<120 mmHg	938 (21.4)	4159 (23.7)	
120–139 mmHg	1935 (44.1)	8187 (46.7)	
≥140 mmHg	1514 (34.5)	5202 (29.6)	
Diastolic blood pressure			<0.001 *
<80 mmHg	1592 (36.3)	7213 (41.1)	
80–89 mmHg	1566 (35.7)	6407 (36.5)	
≥90 mmHg	1229 (28.0)	3928 (22.4)	
Fasting blood glucose			<0.001 *
<100 mg/dL	2824 (64.4)	10,604 (60.4)	
100–125 mg/dL	1140 (26.0)	5126 (29.2)	
≥126 mg/dL	423 (9.6)	1818 (10.4)	
Total cholesterol			0.889
<200 mg/dL	2366 (53.9)	9535 (54.3)	
200–239 mg/dL	1433 (32.7)	5676 (32.4)	
≥240 mg/dL	588 (13.4)	2337 (13.3)	
CCI score			<0.001 *
0	2385 (54.4)	10,115 (57.6)	
1	854 (19.5)	3057 (17.4)	
2	536 (12.2)	1813 (10.3)	
3	298 (6.8)	1119 (6.4)	
≥4	314 (7.2)	1444 (8.2)	
Gastroesophageal reflux disease			<0.001 *
Yes	962 (21.9)	3046 (17.4)	
No	3425 (78.1)	14,502 (82.6)	
H2 blocker	63.03 (123.0)	54.16 (119.9)	<0.001 ^†^
Prescription dates of proton pump inhibitor			<0.001 *
<30 days	451 (10.3)	2679 (15.3)	
≥30 to <365 days	1647 (37.5)	5972 (34.0)	
≥365 days	2289 (52.2)	8897 (50.7)	

Abbreviations: CCI, Charlson comorbidity index; * chi-square test, significance at *p* < 0.05; ^†^ independent *t* test, significance at *p* < 0.05; ^‡^ obesity (BMI, body mass index, kg/m^2^) was categorized as <18.5 (underweight), ≥18.5 to <23 (normal), ≥23 to <25 (overweight), ≥25 to <30 (obese I), and ≥30 (obese II).

**Table 2:** Crude and adjusted odds ratios (95% confidence interval) of prescription dates of proton pump inhibitor for hearing impairment with stratified subgroup according to age and sex.

Characteristics	*N* of Hearing Impairment	*N* of Control	ORs of Hearing Impairment							
	(Exposure/Total, %)	(Exposure/Total, %)	Crude ^†^	*p*-Value	Model 1 ^†,‡^	*p*-Value	Model 2 ^†,^^§^	*p*-Value	Model 3 ^†^	*p*-Value
Total participants (*n* =21,935)										
≥0 to <30 days	451/4387 (10.3%)	2679/17,548 (15.3%)	1		1		1		1	
≥30 to <365 days	1647/4387 (37.5%)	5972/17,548 (34.0%)	1.67 (1.49–1.88)	<0.001 *	1.68 (1.50–1.89)	<0.001 *	1.69 (1.50–1.89)	<0.001 *	1.65 (1.47–1.86)	<0.001 *
≥365 days	2289/4387 (52.2%)	8897/17,548 (50.7%)	1.59 (1.42–1.78)	<0.001 *	1.61 (1.43–1.81)	<0.001 *	1.62 (1.44–1.82)	<0.001 *	1.52 (1.35–1.72)	<0.001 *
Age <70 years old (*n* = 11,910)										
≥0 to <30 days	343/2382 (14.4%)	2073/9528 (21.8%)	1		1		1		1	
≥30 to <365 days	1049/2382 (44.0%)	3752/9528 (39.4%)	1.72 (1.51–1.97)	<0.001 *	1.74 (1.52−1.99)	<0.001 *	1.74 (1.52−1.99)	<0.001 *	1.70 (1.49−1.95)	<0.001 *
≥365 days	990/2382 (41.6%)	3703/9528 (38.9%)	1.67 (1.46−1.93)	<0.001 *	1.70 (1.47−1.95)	<0.001 *	1.70 (1.47−1.96)	<0.001 *	1.60 (1.38−1.86)	<0.001 *
Age ≥70 years old (*n* = 10,025)										
≥0 to <30 days	108/2005 (5.4%)	606/8020 (7.6%)	1		1		1		1	
≥30 to <365 days	598/2005 (29.8%)	2220/8020 (27.7%)	1.51 (1.21–1.90)	<0.001 *	1.51 (1.21–1.89)	<0.001 *	1.52 (1.22–1.91)	<0.001 *	1.49 (1.19–1.87)	<0.001 *
≥365 days	1299/2005 (64.8%)	5194/8020 (64.8%)	1.41 (1.14–1.74)	0.001 *	1.42 (1.15–1.77)	0.001 *	1.43 (1.15–1.78)	0.001 *	1.34 (1.07–1.67)	0.010 *
Men (*n* = 13,255)										
≥0 to <30 days	321/2651 (12.1%)	1964/10,604 (18.5%)	1		1		1		1	
≥30 to <365 days	1058/2651 (39.9%)	3594/10,604 (33.9%)	1.83 (1.60–2.10)	<0.001 *	1.84 (1.60–2.11)	<0.001 *	1.85 (1.61–2.13)	<0.001 *	1.80 (1.57–2.07)	<0.001 *
≥365 days	1272/2651 (48.0%)	5046/10,604 (47.6%)	1.59 (1.39–1.83)	<0.001 *	1.63 (1.41–1.87)	<0.001 *	1.65 (1.43–1.90)	<0.001 *	1.50 (1.30–1.74)	<0.001*
Women (*n* = 8680)										
≥0 to <30 days	130/1736 (7.5%)	715/6944 (10.3%)	1		1		1		1	
≥30 to <365 days	589/1736 (33.9%)	2378/6944 (34.2%)	1.38 (1.12–1.71)	0.002 *	1.39 (1.13–1.71)	0.002 *	1.39 (1.13–1.71)	0.002 *	1.38 (1.12–1.70)	0.003 *
≥365 days	1017/1736 (58.6%)	3851/6944 (55.5%)	1.50 (1.22–1.85)	<0.001 *	1.52 (1.23–1.87)	<0.001 *	1.51 (1.23–1.86)	<0.001 *	1.48 (1.20–1.83)	<0.001 *

Abbreviations: ORs, odds ratios; CCI, Charlson comorbidity index; * conditional logistic regression analysis, significance at *p* < 0.05; ^†^ stratified model for age, sex, income, and region of residence. ^‡^ Model 1 was adjusted for systolic blood pressure, diastolic blood pressure, fasting blood glucose, and total cholesterol. ^§^ Model 2 was adjusted for model 1 plus obesity, smoking, alcohol consumption, and CCI scores. Model 3 was adjusted for model 2 plus gastroesophageal reflux disease and H2 blocker.

**Table 3:** Crude and adjusted odds ratios (95% confidence interval) of prescription dates of proton pump inhibitor for hearing impairment by severity of hearing impairment.

Characteristics	*N* of Hearing Impairment	*N* of Control	ORs of Hearing Impairment							
	(Exposure/Total, %)	(Exposure/Total, %)	Crude ^†^	*p*-Value	Model 1 ^†,^^‡^	*p*-Value	Model 2 ^†,^^§^	*p*-Value	Model 3 ^†^	*p*-Value
Severe hearing loss (*n* = 4075 for hearing impairment, *n* = 16,300 for control)										
≥0 to <30 days	409/4075 (10.0%)	2457/16,300 (15.1%)	1		1		1		1	
≥30 to <365 days	1528/4075 (37.5%)	5502/16,300 (33.8%)	1.70 (1.51–1.92)	<0.001 *	1.71 (1.52–1.93)	<0.001 *	1.72 (1.52–1.94)	<0.001 *	1.69 (1.49–1.90)	<0.001 *
≥365 days	2138/4075 (52.5%)	8341/16,300 (51.2%)	1.60 (1.42–1.81)	<0.001 *	1.63 (1.45–1.84)	<0.001 *	1.64 (1.45–1.86)	<0.001 *	1.55 (1.37–1.76)	<0.001 *
Profound hearing loss (*n* = 312 for hearing impairment, *n* = 1248 for control)										
≥0 to <30 days	42/312 (13.5%)	222/1248 (17.8%)								
≥30 to <365 days	119/312 (38.1%)	470/1248 (37.7%)	1.36 (0.92–2.02)	0.122	1.34 (0.91–1.99)	0.155	1.33 (0.90–1.98)	0.140	1.30 (0.87–1.94)	0.196
≥365 days	151/312 (48.4%)	556/1248 (44.6%)	1.49 (1.00–2.20)	0.048 *	1.39 (0.94–2.08)	0.143	1.35 (0.90–2.03)	0.102	1.26 (0.83–1.91)	0.288

Abbreviations: CCI, Charlson comorbidity index; * conditional logistic regression analysis, significance at *p* < 0.05. ^†^ Stratified model for age, sex, income, and region of residence. ^‡^ Model 1 was adjusted for systolic blood pressure, diastolic blood pressure, fasting blood glucose, and total cholesterol. ^§^ Model 2 was adjusted for model 1 plus obesity, smoking, alcohol consumption, and CCI scores. Model 3 was adjusted for model 2 plus gastroesophageal reflux disease and H2 blocker.

## Data Availability

Releasing of the data by the researcher is not allowed legally. All data are available from the database of National Health Insurance Sharing Service (NHISS) https://nhiss.nhis.or.kr/ NHISS allows access to all of this data for the any researcher who promises to follow the research ethics at some cost. If you want to access the data of this article, you can download it from the website after promising to follow the research ethics.
